# Supplementary figures and images for: Robust Parental Preferences in Mental Health Screening in Youth From a Multinational Online Survey
Source: medRxiv. 2023 Feb 10:2023.02.09.23285610. Preprint. [Version 1] doi: 10.1101/2023.02.09.23285610 (PMC9934784; doi:10.1101/2023.02.09.23285610)

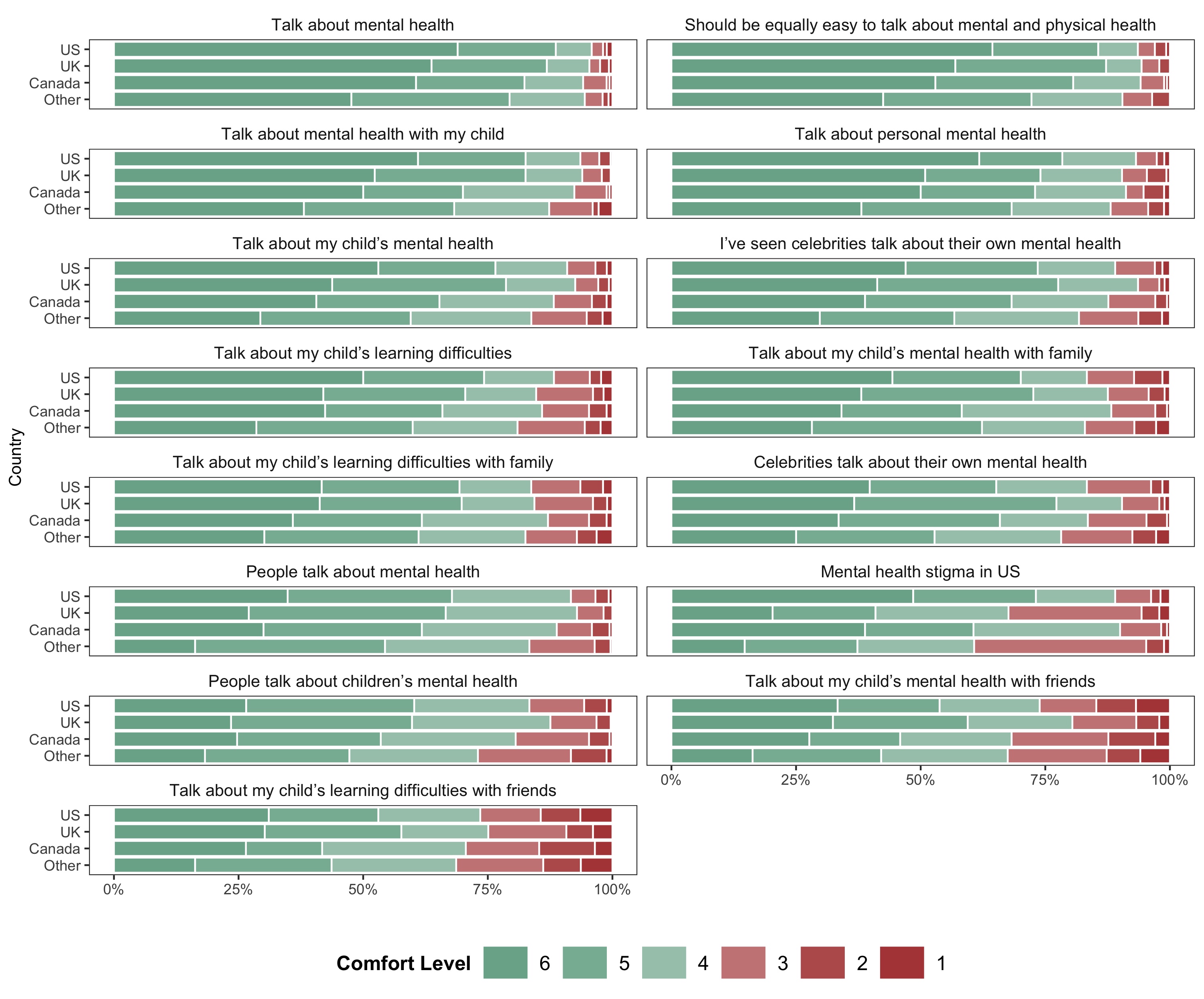

Supplement: Supplement 1 — eFigure 1. Parents’ Willingness to Discuss Mental Health and Learning Disorders. Parents’ comfort levels on various statements about their willingness and/or ability to discuss mental health and learning disorders. The rating scale was a 6-point Likert scale, with 1=Disagree and 6=Agree. [file media-1.jpg]

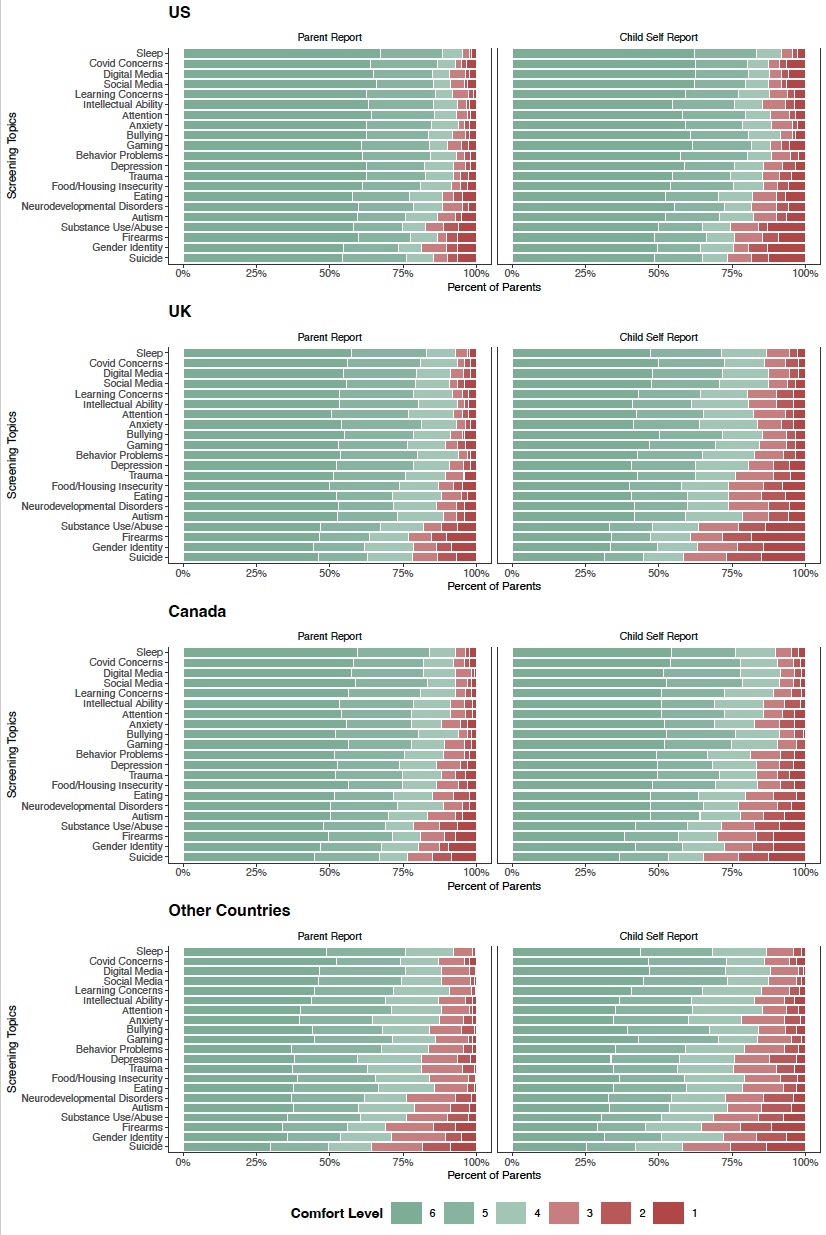

Supplement: Supplement 2 — eFigure 2. Average Parental Comfort Levels by Country. * p<.05, **p<.01, ***p<.001. Parental comfort levels of parent report and child self-report for various screening topics by country sample (US: n=265; UK: n=282; Canada: n=171; Other Countries: n=254). Topics are ordered according to the entire sample (N=972). Comfort levels ranged from 1 (not comfortable) to 6 (comfortable). [file media-2.jpg]
